# COVID-19 and Toll-Like Receptor 4 (TLR4): SARS-CoV-2 May Bind and Activate TLR4 to Increase ACE2 Expression, Facilitating Entry and Causing Hyperinflammation

**DOI:** 10.1155/2021/8874339

**Published:** 2021-01-14

**Authors:** Mohamed M. Aboudounya, Richard J. Heads

**Affiliations:** Department of Cardiology, The Rayne Institute, St Thomas' Hospital, British Heart Foundation Centre of Research Excellence, School of Cardiovascular Medicine and Sciences, King's College London, UK

## Abstract

Causes of mortality from COVID-19 include respiratory failure, heart failure, and sepsis/multiorgan failure. TLR4 is an innate immune receptor on the cell surface that recognizes pathogen-associated molecular patterns (PAMPs) including viral proteins and triggers the production of type I interferons and proinflammatory cytokines to combat infection. It is expressed on both immune cells and tissue-resident cells. ACE2, the reported entry receptor for SARS-CoV-2, is only present on ~1-2% of the cells in the lungs or has a low pulmonary expression, and recently, the spike protein has been proposed to have the strongest protein-protein interaction with TLR4. Here, we review and connect evidence for SARS-CoV-1 and SARS-CoV-2 having direct and indirect binding to TLR4, together with other viral precedents, which when combined shed light on the COVID-19 pathophysiological puzzle. We propose a model in which the SARS-CoV-2 spike glycoprotein binds TLR4 and activates TLR4 signalling to increase cell surface expression of ACE2 facilitating entry. SARS-CoV-2 also destroys the type II alveolar cells that secrete pulmonary surfactants, which normally decrease the air/tissue surface tension and block TLR4 in the lungs thus promoting ARDS and inflammation. Furthermore, SARS-CoV-2-induced myocarditis and multiple-organ injury may be due to TLR4 activation, aberrant TLR4 signalling, and hyperinflammation in COVID-19 patients. Therefore, TLR4 contributes significantly to the pathogenesis of SARS-CoV-2, and its overactivation causes a prolonged or excessive innate immune response. TLR4 appears to be a promising therapeutic target in COVID-19, and since TLR4 antagonists have been previously trialled in sepsis and in other antiviral contexts, we propose the clinical trial testing of TLR4 antagonists in the treatment of severe COVID-19. Also, ongoing clinical trials of pulmonary surfactants in COVID-19 hold promise since they also block TLR4.

## 1. Introduction

The SARS-CoV-2 virus has caused a worldwide pandemic in 2020, infecting millions of people and leading to a global health crisis [1]. COVID-19, the disease caused by SARS-CoV-2, not only leads to serious lung inflammation, pneumonia, and acute respiratory distress syndrome (ARDS) in vulnerable individuals, but also causes injury to multiple organs including cardiac damage and renal injury and can lead to death [2, 3]. In severe cases, infected patients can exhibit a “cytokine storm” or hyperinflammation in the form of excessive production of proinflammatory cytokines, which is usually associated with negative outcomes [4]. There have been devastating health consequences worldwide, with high mortality and morbidity. The disease is still a puzzle, and many questions on its pathology remain unanswered. In 2002, the world had witnessed an outbreak of SARS in China caused by the SARS-CoV-1 (also known as SARS-CoV), which is phylogenetically and genetically similar to the novel SARS-CoV-2 [5]. Infected patients displayed similar pneumonia symptoms with a diffused alveolar injury, which leads to ARDS [5]. Findings from studies on SARS-CoV-1 and other viruses infecting humans can provide clues to the pathophysiology of SARS-CoV-2.

Like SARS-CoV-1, the novel SARS-Cov-2 virus contains a single-stranded RNA of ~30 kilobases, which encodes for multiple structural and nonstructural proteins, surrounded by an outer envelope [5]. The genome has been reported to be more than 80% identical to that of the previous SARS-CoV-1 [5, 6]. The structural proteins include the spike (S) protein, the membrane (M) protein, the envelope (E) protein, and the nucleocapsid (N) protein [5, 7]. Both SARS-CoV-2 and SARS-CoV-1 use angiotensin converting enzyme 2 (ACE2) as an entry receptor in humans, via binding of their outer surface spike S protein [8, 9]. The S protein of SARS-CoV-2 is also primed by a human cellular protease (transmembrane protease, serine2 (TMPRSS2)) [8]. Cells with ACE2 expression, such as type II alveolar cells in the lungs, are susceptible to SARS-CoV-2 infection [10, 11].

To successfully fight viral infections, the body relies on the innate and adaptive immune systems. The innate immune system is the first line of defence and is responsible for recognizing certain structurally conserved components of a virus: to mount an early production of interferons as well as proinflammatory cytokines and chemokines. These interferons are antiviral molecules that alert neighbouring cells to the viral presence and suppress the viral replication efficiency [12]. Interferons and other cytokines recruit immune cells (e.g., natural killer cells, neutrophils, and monocytes) for the subsequent cellular cytotoxic and adaptive immune responses. Specialised B and T lymphocytes are part of the adaptive immune system, which is aimed at producing specific neutralising antibodies against the virus and developing long-lasting memory against it [12]. However, since neutralising antibodies against SARS-CoV-2 have been shown to decline over a period of only 3 months postinfection in humans [13], there is a need to adopt a parallel treatment strategy that involves the innate immune system, besides antiviral drugs.

TLR4 is a remarkable pattern recognition receptor (PRR) that belongs to the innate immune system. It recognizes multiple pathogen-associated molecular patterns (PAMPs) from bacteria, viruses, and other pathogens [14]. In addition, it recognizes certain damage-associated molecular patterns (DAMPs) such as high mobility group box 1 (HMGB1) and heat shock proteins (HSPs) released from dying or lytic cells during host tissue injury or viral infection [12, 15]. TLR4 is not only expressed on the cell surface of immune cells such as macrophages and dendritic cells where it plays a role in the regulation of acute inflammation, but also on some tissue-resident cell populations, for cell defence in case of infection and/or to regulate their fibrotic phenotype in cases of tissue damage (e.g., [16–19]). The archetypal PAMP agonist for TLR4 is the gram-negative bacterial lipopolysaccharide [20]. In a simplistic view, activation of TLR4 by pathogenic components leads to the production of proinflammatory cytokines via the canonical pathway and/or the production of type I interferons and anti-inflammatory cytokines via the alternative pathway. There are other TLRs, such as TLR3 which is anchored to intracellular endosomes and recognizes double-stranded RNA (dsRNA) motifs from invading pathogens, after they have invaded the cells [21]. In contrast, TLR4 is present both at the cell surface (main site) where it recognizes viral proteins before they enter the cell and also in endosomes when its alternative signalling pathway is triggered [22]. TLR4 is important in initiating inflammatory responses, and its overstimulation can be detrimental leading to hyperinflammation. Dysregulation of TLR4 signalling has been shown to play a role in the initiation and/or progression of various diseases, such as ischaemia-reperfusion injury, atherosclerosis, hypertension, cancer, and neuropsychiatric and neurodegenerative disorders (see [23–28]). Moreover, TLR4 is also important in the induction of the host immune response against infectious diseases such as bacterial, fungal and viral infections, and malaria [29].

Here, we review and link together evidence from several studies and find that when viewed together, they shed light on the complex “jigsaw puzzle” of COVID-19 pathophysiology as relating to virus entry, symptoms, and inflammatory signalling. For instance, (1) evidence that TLR4 has the strongest protein-protein interaction with the spike glycoprotein of SARS-CoV-2 compared to other TLRs [30], together with (2) evidence that SARS-COV-2 strongly induces interferon-stimulated gene (ISG) expression in an immunopathogenic context in the respiratory tract [31]; (3) evidence that ISG activation results in increased expression of ACE2 [32] and (4) evidence that pulmonary surfactants in the lung prevent viral infection by blocking TLR4 [33] suggest a possible mechanism in which the virus may be binding to and activating TLR4 to increase expression of ACE2 which promotes viral entry. This is supported by the fact that ISGs are downstream of TLR4-interferon signalling, thereby linking these phenomena.

**Figure 1:**
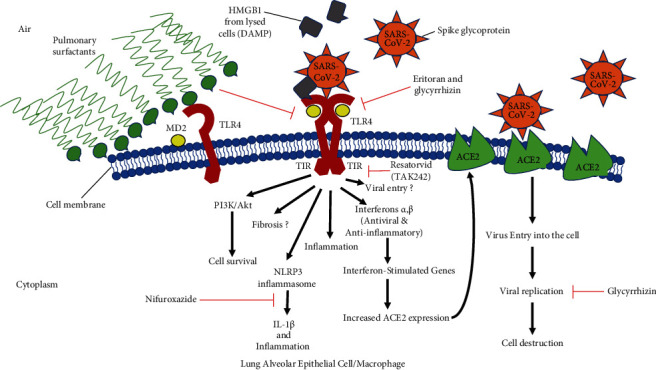
Graphical abstract. Note that less pulmonary surfactant secretion leads to exposed TLR4 receptors on alveolar cells. HMGB1 released from lysed cells can also activate TLR4 causing excessive inflammation and fibrosis. The same model applies to a cardiac cell, except that there would be no pulmonary surfactants, and instead of the outside air, it would be the extracellular matrix. In the heart, TLR4 activation by the SARS-CoV-2 virus and/or DAMPs released from infected, necrotic cells or even upregulated at sites of injury may also cause abnormal signalling towards the canonical proinflammatory pathway rather than the alternative anti-inflammatory pathway. This would cause viral myocarditis. TLR4 activation also decreases the contractility of cardiomyocytes. In addition, SARS-CoV-2 may activate TLR4 to increase PI3K/Akt signalling in infected cells, preventing apoptosis and thus increasing time for viral replication. Aberrant inflammatory signalling could also be extended to other tissues expressing TLR4, such as the skin and kidney, where the virus would therefore cause multiple-organ injury.

Moreover, studies have focussed on the finding that SARS-CoV-2 infects type II alveolar cells in the lungs simply because they express ACE2. However, since these particular type II alveolar cells are the ones that produce pulmonary surfactants [34], it means that infected patients would have less pulmonary surfactants leading to alveolar collapse and difficulty breathing. Pulmonary surfactants reduce the surface tension in the lungs at the air/liquid interface of the tissue, allowing lung expansion (compliance), gaseous exchange, and alveolar stability [35, 36]. They also antagonize or block TLR4 receptors on the lung cells [37, 38]. Firstly, this could explain some of the respiratory symptoms, ARDS, and why ventilators may not be wholly effective in COVID-19 patients; i.e., it resembles neonatal respiratory distress syndrome (NRDS) in new-born babies, who are born prematurely with low levels of pulmonary surfactants. Secondly, it also means that TLR4 on the lung cells is being exposed for the virus to bind to, due to reduced pulmonary surfactants. Hence, a possible model for the interaction of SARS-CoV-2 and TLR4 is outlined in Section 11 and the graphical abstract (Figure 1) in which SARS-CoV-2 may activate TLR4 in the heart and lungs to cause aberrant TLR4 signalling in favour of the proinflammatory MyD88-dependent (canonical) pathway rather than the alternative TRIF/TRAM-dependent anti-inflammatory and interferon pathway. Therefore, this probably contributes to the acute lung injury, myocarditis, cardiac complications, and other severe inflammatory complications such as the hyperinflammation observed in patients with severe COVID-19 disease. From a therapeutic perspective, interesting recent developments include the use of pulmonary surfactants administered by endotracheal intubation to treat COVID-19, which is the focus of a number of recent clinical trials. We also briefly review the proposed use of TLR4 antagonists as antiviral treatments, including Eritoran, Resatorvid (CLI-095/TAK242), and glycyrrhizin, as well as another compound, nifuroxazide, that interrupts TLR4 signalling. This review highlights a potential role of TLR4 in SARS-CoV-2 entry mechanisms and the subsequent severe inflammatory complications with a focus on injury to the lungs and heart, since they are the major causes of mortality from COVID-19.

## 2. COVID-19 and Mortality from Lung and Heart Complications

COVID-19 causes acute lung injury (ALI) and ARDS, which leads to pulmonary failure and subsequent death [5]. Zhang et al. reported the clinical characteristics of 82 fatalities from COVID-19 from Wuhan local hospital records [39]. They found that respiratory failure accounted for 69.5% of deaths and was the leading cause, followed by sepsis/multiorgan failure at 28.0%, cardiac failure at 14.6%, haemorrhage at 6.1%, and renal failure at 3.7%. Another study by Ruan et al. analysed 68 deaths from Wuhan and found that 53% of patients died of respiratory failure, 33% died of both respiratory failure with myocardial damage/heart failure, and 7% died of myocardial damage/heart failure alone, while the remaining 7% died of an unknown cause [40]. Furthermore, it is critical to note that Zhang et al. found respiratory and cardiac damage in 100% and 89% of patients, respectively, followed by haemorrhagic (80.5%), hepatic (78.0%), and renal damage (31.7%) [39]. Therefore, we shall focus in this review on injury to the lungs and the heart and their relation to TLR4 since they account for most of the fatalities.

For the lungs, SARS-CoV-2 infects and damages the type II alveolar cells, causing severe injury to the alveoli, destruction of the lung alveolar architecture, and the formation of hyaline membranes, thus impairing lung function and gaseous exchange [41]. This leads to breathlessness and respiratory failure, which is further escalated by severe inflammation in the lungs. For the heart, as reviewed by Akhmerov et al., cardiac injury by SARS-CoV-2 can present in the form of (1) elevations of cardiac injury biomarkers (cardiac troponin and brain-type natriuretic peptide), (2) arrhythmias, (3) myocardial infarction/acute coronary syndrome (ACS), and (4) heart failure [42]. They also reported that cardiac injury can result via direct or indirect mechanisms. The direct mechanism involves viral infiltration into myocardial tissue, i.e., a direct infection, resulting in cardiomyocyte death and inflammation. The indirect mechanism is secondary to respiratory failure and hypoxemia which leads to cardiac stress and hypoxia-related myocyte injury. Cardiac inflammation as a result of severe systemic hyperinflammation is also included under the indirect mechanism [42], but deserves to be a third mechanism in its own right, since it might involve sepsis, TLR4 activation, and/or the cytokine storm (also called “immune-mediated cytokine release syndrome”) [43]. At approximately two and a half months after COVID-19 diagnosis, ongoing myocardial inflammation was found in about 60% of already-recovered patients, independent of preexisting conditions [44]. Therefore, this implicates the involvement of inflammatory receptors such as TLR4.

## 3. The Reported Entry Mechanisms for Direct Lung and Cardiac Infection by SARS-CoV-2

For the previous SARS-CoV-1, angiotensin converting enzyme 2 (ACE2) provided the main entry site for the virus into human hosts, being highly expressed in lung alveolar cells [45]. Other studies have also reported that lung cells including type II pneumocytes (which express ACE2) and ciliated cells of the airway epithelium are the primary targets of SARS-CoV-1 and IAV infection in the lung [46, 47]. More recently, Zou et al. undertook singe-cell RNA-seq data analysis and revealed that the average proportion of ACE2-positive alveolar type II cells from eight individuals was approximately 1%, which was defined by the authors as sufficient for a potential high risk of viral infection [10]. The respiratory epithelial cells from the respiratory tract samples contained approximately 2% of ACE2 positive, which again was regarded as high risk. Thus, it appears that the alveolar type II cells and respiratory epithelial cells exhibit ACE2 expression, and these are what SARS-CoV-2 mainly infects in the lungs. Fu et al. also showed that human ACE2 expression in the lungs is very low, compared to other organs such as the heart, kidneys, and small intestines [48]. Respiratory symptoms are more severe in patients with an underlying cardiovascular disease (CVD), which might be explained by the increased ACE2 expression in those patients compared with healthy individuals, especially if they take ACE inhibitor or RAAS inhibitor medications [2, 49, 50]. However, this view has been challenged (see below).

For the heart, reviews have proposed direct viral entry via spike protein interacting with the specific ACE2 receptors on cardiomyocytes and thereby toxicity in host cells [43, 51], although one commentary regarded it as a “potential mechanism” in the presence of limited data, given that ACE2 “is highly expressed in the heart and lungs” [49, 52]. scRNA-seq data showed more than 7.5% myocardial cells were positive for ACE2 expression putting the heart at risk in the presence of viraemia [10]. ACE2 was found to be expressed in multiple cells in the heart: cardiomyocytes and mural cells, particularly pericytes which may act as the initial target cells and result in endothelial and microvascular dysfunction [53, 54]. ACE2 is also expressed but at lower levels, in fibroblasts, endothelial cells, and leucocytes [53]. Thus, given that ACE2 is expressed at much lower levels in the lungs than the heart and since one study involving 58 patients found that ACE inhibitors/angiotensin II receptor blockers have no significant effect on the outcome and prognosis of COVID-19 patients with hypertension [55], this raises questions about the evidence for the correlation between ACE2 levels and infectivity of SARS-CoV-2.

## 4. Cardiovascular Comorbidities, ACE2, and TLR4

The majority of patients who died from COVID-19 had cardiac comorbidities (76.8%), including hypertension (56.1%), coronary heart disease (20.7%), followed by diabetes (18.3%), cerebrovascular disease (12.2%), and cancer (7.3%) [39]. Ruan et al. also found that patients with cardiovascular diseases have a significant risk of death when infected with SARS-CoV-2 [40]. Thus, it appears that having an actual underlying CVD is a major risk factor for death, not simply due to tissue expression of ACE2, although ACE2 upregulation is associated with heart failure or ischaemic cardiomyopathy (see [52] and references therein), and ACE2 elevation occurred in cardiomyocytes of patients with heart disease compared with healthy controls [53]. Many of these patients might not necessarily be taking ACE inhibitors; i.e., they would probably have upregulation of other receptors in the heart due to their underlying CVD. In fact, in another study, the use of ACE inhibitors/angiotensin II receptor antagonists had no significant effect on the morbidity or mortality of COVID-19 [56]. Furthermore, Tian et al. found that there is a difference in the receptor-binding domain (RBD) of the spike protein between SARS-CoV-1 and SARS-CoV-2 [57], which means that other receptor(s) might be implicated. Moreover, that ACE2 is expressed more in the myocardium than in the lungs, when COVID-19 primarily or initially affects the lungs, raises speculations of possible different entry mechanisms.

This raises the question, is it only ACE2 that the spike protein of SARS-CoV-2 binds to or is there also another receptor involved, such as TLR4 which is known to promote cardiac hypertrophy, myocardial inflammation, and fibrosis [58–60]? Moreover, activation of TLR4 on platelets whether by PAMPs (viraemia and LPS) or DAMPs induces a prothrombotic and proinflammatory state [61], which provides a potential explanation for the thrombotic events (such as MI) observed in COVID-19 patients. Computationally, the spike protein binds stronger to TLR4 than ACE2 [30], and if there is a secondary bacterial infection in COVID-19 (which accounted for 10% of ICU patients in one study [2]), TLR4 would also be activated by gram-negative bacterial LPS. In fact, TLR4 is upregulated in the infarcted and noninfarcted myocardium following ischaemia/reperfusion injury and in heart failure [62, 63]. Even if the virus infects cardiomyocytes via ACE2 only, the subsequent immune-mediated myocardial injury and inflammation is likely mediated via TLR4 due to the DAMPs released from the lysed cardiomyocytes. The following sections combine several pieces of evidence illustrating that it could play a prominent role in COVID-19 pathophysiology.

## 5. TLR4 Expression in the Lungs and the Heart

TLR4 is expressed mainly on cells of the immune system, including macrophages, dendritic cells, and monocytes [64]. These could be tissue resident in the lungs or heart or migratory following infection or cell death. In the lungs, TLR4 is also expressed at low basal levels in alveolar cells, including type I and II cells, and bronchial epithelial cells, but interestingly, TLR4 expression and sensitisation (to LPS) increase upon inflammatory insults, infiltration, or coculturing with macrophages or viral infections such as RSV [19, 65–68]. It is also expressed on alveolar macrophages and lung fibroblasts [69, 70]. Activation of TLR4 in the lungs leads to an intense inflammatory response with Nod-like receptor protein 3 (NLRP3) inflammasome activation and secretion of proinflammatory cytokines including TNF-*α*, IL-1*β*, and IL-18. TLR4 signalling was identified as a key pathway of acute lung injury [67, 71–73]. Inhibition of the NLRP3 inflammasome signalling pathway downstream of TLR4 attenuates LPS-induced acute lung injury [74, 75]. TLR4 can also contribute to lung fibrosis [76].

In the heart, TLR4 is expressed on cardiomyocytes, cardiac fibroblasts, and cardiac macrophages [77–79]. In fact, TLR4 is the most abundantly expressed TLR in the heart, followed by TLR2 and TLR3 [80]. TLR4 plays an important role in the regulation of myocardial function, fibroblast activation, and acute inflammation by immune cells. TLR4 activation by LPS on cardiomyocytes leads to subsequent reduction in myocardial contractility [77, 81], and the predominant view in the literature is that TLR4 activation on cardiac structural fibroblasts and cardiac macrophages leads to a profibrotic and proinflammatory response, respectively [78, 82]. Furthermore, LPS causes septic cardiomyopathy via TLR4 activation [83], which is of relevance since sepsis—whether viral or bacterial—is implicated in severe cases of COVID-19.

## 6. TLR4 Signalling

TLR4 is a single transmembrane protein with a horseshoe-shaped extracellular domain that contains many leucine-rich repeats [84, 85]. It is mainly present on the cell surface membrane, while its cytoplasmic tail contains an intracellular Toll/interleukin-1 receptor (TIR) domain [84, 85]. As mentioned previously, TLR4 is activated by its typical ligand, LPS. It is also activated by DAMPs released from lytic or necrotic cells such as HMGB1 and HSPs or upregulated at sites of tissue injury and chronic inflammation such as the alternatively spliced fibronectin-extra domain A (Fn-EDA) and other ECM-derived DAMPs including low molecular weight hyaluronan (LMWHA) and sulphated proteoglycans [15, 17, 23, 86]. These DAMPs drive the expression of fibroinflammatory genes at sites of wound healing, often leading to maladaptive remodelling and fibrosis [87]. More relevantly, TLR4 is activated by viral PAMPs to initiate an innate immune and inflammatory response. TLR4 uses an accessory protein called MD2 for the recognition of LPS and viral proteins; MD2 initially binds to TLR4 within the cell and is also necessary for the correct trafficking of TLR4 to the cell surface [85]. The coreceptor, CD14, also plays a role in bringing LPS to TLR4 and in endocytosis [85, 88].

**Figure 2:**
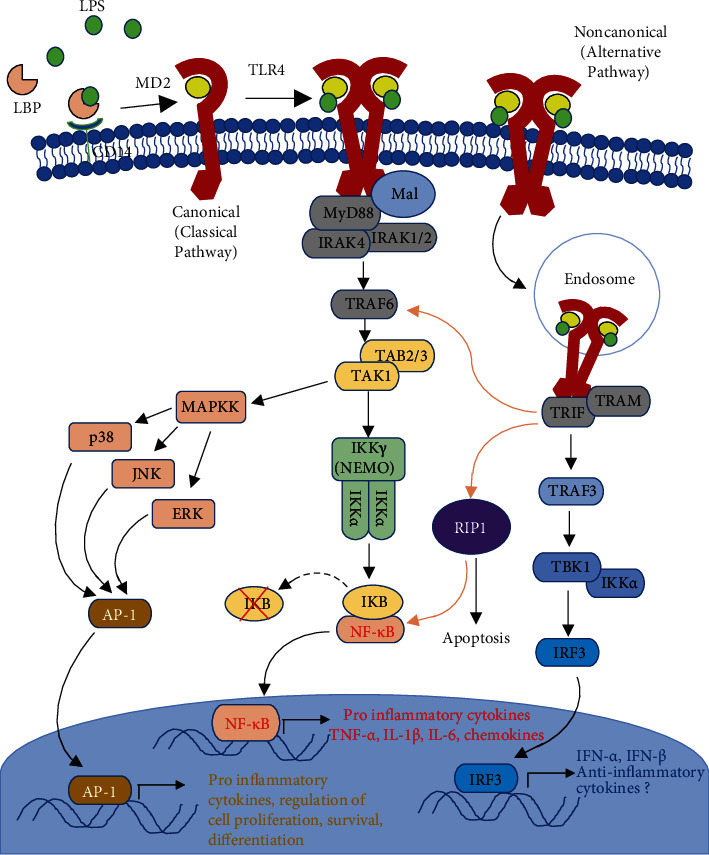
Overview of the main TLR4 signalling pathways. TLR4 can be activated by LPS (classical PAMP), DAMPs, or viral PAMPs. LPS is also picked up by the LPS-binding protein (LBP) present in the blood and in extracellular fluid in tissues. LPS is transferred from LBP to the coreceptor CD14. Recognition by MD2 and TLR4 binding results in the nuclear induction of transcription factors NF-*κ*B and AP-1 via the MyD88-dependent pathway involving IRAKs, TABs, TAK, MAP kinases, and IKK isoforms. This results in the transcription of proinflammatory cytokines and regulators of cell proliferation, survival, and differentiation. In fibroblasts, this pathway mediates myofibroblast differentiation and results in CTGF and collagen production [100], while the alternative pathway is endosomal and involves TRIF and TRAM complex formation at the TIR domain. The alternative pathway results in the expression of anti-inflammatory cytokines, interferons *α* and *β* regulated by interferon regulatory factor 3 (IRF3), in addition to some NF-*κ*B nuclear translocation. “Cross-talk” activation between the two pathways is indicated by brown arrows. Adaptors and cascade proteins are usually in homodimers, like the TLR4 receptor [22], but were omitted here for simplicity.

The canonical pathway of TLR4 signalling culminates in the activation of the transcription factor NF-*κ*B, which induces the expression of proinflammatory cytokines and chemokines, especially TNF-*α*, IL-1*β*, and IL-6 [89, 90]. Ligand binding to TLR4 induces the dimerization of the receptor, followed by the differential association of its TIR domains with the MyD88 (myeloid differentiation factor 88) adaptor protein; hence, this canonical pathway is also called the MyD88-dependent pathway (Figure 2) [85, 89]. MyD88 in turn recruits and activates adaptor proteins IL-1 receptor-associated kinases IRAK4 and IRAK1 followed by a sequential activation cascade involving proteins such as TRAF6, TAK1, and TAB2 (see Figure 2) [85, 89]. Eventually, activated TAK1 leads to the activation of I*κ*B kinase (IKK), which phosphorylates and degrades the inhibitory molecule I*κ*B. This allows dimerization and nuclear translocation of the p65 and p50 subunits of NF-*κ*B allowing it to induce the transcription of proinflammatory cytokines and other inflammatory mediators [84, 85, 89]. TAK1 can also activate the ERK1/2 mitogen-activated protein kinases (MAPK), JNK, and p38, which phosphorylate and activate of the heterodimeric transcription factor AP-1 (i.e., cFos, cJun, and ATF2) [85, 89]. AP-1 regulates some processes involving cell proliferation, survival, differentiation, and apoptosis as well as the expression of proinflammatory cytokines [89, 91].

However, TLR4 signalling can also result in an alternative, noncanonical pathway that is MyD88 independent [89, 92]. This involves TLR4 recruiting the adaptors TRIF and TRAM and concludes with the production of type I interferons [84, 90]. TRIF and TRAM interact with TRAFs which leads to the recruitment of kinases TBK1 and IKK*ε* [84, 90]. These mediate the phosphorylation of the transcription factor interferon regulatory factor 3 (IRF3), producing type I interferons [90]. The current view is that this pathway might occur when the receptor is present intracellularly on endosomes [22, 88, 93]. One “cross-talk” mechanism between the two pathways is that TRIF interacts with receptor-interacting protein-1 (RIP1) which is directly responsible for the activation of NF-*κ*B and IFN-*β* and induces apoptosis [64, 89]. Thus, this pathway might not only lead to the weaker and later activation of NF-*κ*B compared to canonical activation in immune cells, but also to mainly IRF3 activation [84, 90]. This alternative signalling pathway is believed to confer partial anti-inflammatory responses, especially that it results in type I interferon production. Type I interferons (IFN-*α* and *β*) are antiviral cytokines that provide first lines of defence against viral infections, alerting other cells of the virus infection and reducing protein synthesis inside the cells [94]. However, interferon-*β* (IFN-*β*) that is produced by fibroblasts is also immunomodulatory and anti-proliferative and downregulates the expression of proinflammatory cytokines and MHC class II molecules in antigen presenting cells, while upregulating the expression of anti-inflammatory cytokines, such as IL-10 [95, 96]. Besides IFN-*β*, the transcription factor IRF3 itself can also activate the transcription of IL-1 receptor antagonist (IL-1Ra) and the anti-inflammatory IL-10 in microglia cells, besides other proinflammatory chemokines, such as CCL5 [97].

Interferons also induce the expression of interferon-stimulated genes (ISGs), which exert antiviral effects [94]. Secreted type I interferons bind to interferon alpha-beta receptors (IFNARs) that are ubiquitously expressed on cell membranes, to activate the Jak/Stat (Janus-kinase and signal transducer and activator of transcription) signalling pathway, which induces the transcription of a wide variety of ISGs [98]. ISG products then block viral replication at different phases of its replication cycle [98]. Notably, IRF3 can even stimulate some ISGs directly; bypassing IFNs [99].

However, ACE2 has recently been found to be an ISG in airway epithelial cells [32]. This makes sense because ACE2 is anti-inflammatory and is generally protective in the lungs and in fact suppresses the LPS-TLR4 inflammatory pathway in LPS-induced ALI in mice [101, 102]. This could also be viewed as a kind of negative feedback mechanism to regulate TLR4 signalling. Note that in the heart, ACE2 is cardioprotective; it converts angiotensin II to angiotensin-(1-7), which has anti-inflammatory and vasodilatory properties [50, 103, 104].

In structural fibroblasts, TLR4 activation also leads to the induction of a myofibroblast phenotype that secretes collagens and other profibrotic mediators [87, 105]. LPS-TLR4 signalling can also activate the NLRP3 inflammasome in the lungs and heart, which results in the secretion of IL-1*β* and IL-18 and inflammation [75, 106–108].

A third TLR4 signalling pathway can also occur. Glucan phosphate, a TLR4 ligand, leads to tyrosine phosphorylation and dissociation of the intracellular domain of TLR4 from MyD88, and instead, its binding to PI3K [89]. PI3K would then phosphorylate Akt (also known as protein kinase B), which inhibits cardiac myocyte apoptosis and promotes survival [109].

## 7. Viral Binding to TLR4

Respiratory syncytial virus (RSV) fusion protein F, Ebola virus (EBOV) glycoprotein, dengue virus nonstructural protein 1 (DENV-NS1), vesicular stomatitis virus glycoprotein (VSV G), and the envelope protein of mouse mammary-tumour virus (MMTV) all bind TLR4 (see [23, 84, 110] and references therein). These proteins cause TLR4 activation, to induce an inflammatory response during acute viral infection. The commonality between these proteins is that they are all membrane-associated glycoproteins that are exposed on the surface of the viral particles. All of them, except DENV-NS1, mediate fusion with the host cell membranes through the hydrophobic fusion peptide (part of the protein that facilitates binding) or a receptor-binding domain (RBD) for MMTV that contains hydrophobic phenylalanine residues [110, 111]. They also appear to activate TLR4 through MD2 binding, like LPS (LPS is hydrophobic) ([110] and references therein). These sound like features that match exactly those of the spike glycoprotein of SARS-CoV-2, being on the virus surface and mediating fusion with host membranes via ACE2.

For influenza A virus (IAV), the mechanisms relating to TLR4 binding/signalling appear to be different, mediated via host DAMPs released from infected cells. Host DAMPs including HMGB1 and oxidized phospholipids are detected in the lungs of patients with severe IAV [71, 110, 112].

## 8. Evidence for SARS-CoV-1 and SARS-CoV-2 Proteins Binding to TLR4 or Causing TLR4 Activation via DAMP-Related Mechanisms

### 8.1. Evidence for Direct Binding of Viral Proteins

The binding of the spike protein to TLR4 may be involved in SARS-CoV-2 entry into human cells and/or initiating the cytokine storm that affects multiple organs, a serious complication which occurs in the severe stages of COVID-19. Choudhury and Mukherjee undertook an *in silico* study and found that the spike glycoprotein of SARS-COV-2 had the strongest protein-protein interaction with TLR4, compared to other TLRs [30]. Molecular docking studies showed that it had a respective binding energy of -120.2 (the more negative the energy, the better the ligand) with the extracellular domain of TLR4, via hydrogen bonding and hydrophobic interactions [30]. The study appears valid because they found that the spike glycoprotein also binds strongly to the known ACE2 entry receptor from both human and bat origins (-29.2 and -44.4 binding energies, respectively) and was phylogenetically similar to bat coronavirus. Indeed, we deduce that the spike glycoprotein-TLR4 interaction is stronger than the spike glycoprotein-ACE2 interaction, which is a critical finding that must be exploited. This shows that TLR4 at the cell surface is more likely to be involved in recognising molecular patterns from SARS-CoV-2 to trigger inflammatory responses, compared to other TLRs. TLR4 may also be involved in promoting viral cell entry in other organs, such as the skin where SARS-CoV-2 has caused complications in some patients and which has a relatively low ACE2 expression [48]; TLR4 is also expressed on skin fibroblasts [105]. This adds evidence to the possibility that TLR4 might be used by the virus to gain entry into the cells, especially that TLR4 does not need TMPRSS2.

For SARS-CoV-1, there is evidence of direct recognition (or binding) of a viral protein by TLR4. TLR4-/- mice are more susceptible to SARS-CoV-1 than wild-type mice with higher viral titers [113], which means that there was impairment in the innate immune response due to the lack of TLR4, and hence difficulty in fighting the virus. This provides evidence that TLR4 is important in recognizing or sensing a certain viral PAMP of SARS-CoV-1 to initiate a protective antiviral response, regardless of the viral titers in that study. SARS-CoV-1 is only similar, and not identical, to the SARS-CoV-2, which implies differences in their virulence and pathological entry mechanisms. In fact, wild-type mice cleared the SARS-CoV-1 virus by day 7 postinfection. Nevertheless, the TLR4-/- mice experienced only transient weight loss with no mortality in response to an infection and also cleared the virus by day 7 postinfection (despite having higher viral titers than wild-type mice at days 2 and 4 postinfection) [113], which has good implications for trying a TLR4 antagonist in SARS-CoV-2 infections. An antagonist would have a pharmacokinetic profile allowing some receptor activation, i.e., different from total ablation.

Enayatkhani et al. also used in silico studies and evaluations to design a novel vaccine candidate against COVID-19 [114]. The vaccine contained epitope-rich domains from 3 antigenic proteins intended to elicit a humoral and cell-mediated immune response: the nucleocapsid, ORF3a (Open Reading Frame 3a), and membrane protein; it was called the “NOM” vaccine. Through molecular docking studies, the best 3D predicted model of the vaccine was found to bind well to TLR4 (innate immune system receptor) and also to HLA-A^∗^11 : 01 (cellular immune system receptor)—the models were stable during stimulation time [114]. Although this was designed, and multiple amino acids from the 3 epitopes in the NOM protein are contributing to the binding to TLR4, it shows that components of SARS-COV-2 are binding well to TLR4 and raises further questions. More recently, several other *in silico* studies have designed multiepitope vaccines using SARS-CoV-2 spike glycoprotein constructs/subunits, and they are found to bind TLR4 using molecular docking studies (see [115–119]).

An earlier study by Wang and Liu showed that the membrane M protein of SARS-CoV-1 functions as a novel cytosolic pathogen-associated molecular pattern (PAMP) to promote IFN-*β* induction via a toll-like receptor-related TRAF3-independent mechanism [120]. This is intriguing because TRAF3 is part of the TLR-mediated (particularly TLR4 and TLR3) alternative (endosomal) pathways, which are responsible for the production of IFNs [120]. This can be extrapolated to SARS-CoV-2, where intracellularly, its M protein may be inducing TLR4-dependent TRAF3-independent IFN-*β* production.

### 8.2. Indirect Evidence from Downstream Signalling Mechanisms and TLR4 Expression

There is evidence from *in vivo* studies that a certain ligand in SARS-CoV-1 infection activates both TLR4 downstream signalling pathways: the canonical MyD88-dependent pathway and the alternative TRIF/TRAM-dependent pathway. This makes sense, because in order to mount the cell's powerful innate immune response against the virus, the host cells need both: NF-*κ*B to trigger the transcription of proinflammatory cytokines and chemokines and IRF3 to express the antiviral and anti-inflammatory interferons that in turn, induce the transcription of ISGs.

MyD88-/- mice infected with SARS-CoV-1 were shown to have higher mortality, weight loss, and high viral loads compared to controls. Therefore, MyD88 is required for protection from lethal infection of SARS-CoV-1, especially that it is a main adaptor protein for multiple TLRs, not only TLR4 [121]. In addition, TRAM-/- mice are more susceptible to SARS-CoV-1 infection, but experience only transient weight loss with no mortality in response to infection [113]. However, TRIF-/- mice were highly susceptible to SARS-CoV-1 infection, displaying increased weight loss, mortality, reduced lung function, increased lung pathology, and higher viral titers compared to their control counterparts [113]. This is due to TRIF being the adaptor for both TLR4 and TLR3 [12]. Hence, Totura et al. illustrate that MyD88-independent, TRIF/TRAM-dependent signalling is necessary to fight the viral infection [113].

Overall, MyD88-negative and TRIF-negative mice had similar mortality, weight loss, and viral loads, which shows that a balance is needed between both signalling pathways for a proper innate immune response [113, 121]. We can confidently extrapolate the above findings in Sections 8.1 and 8.2 from SARS-CoV-1 to SARS-CoV-2; hence, we propose that SARS-CoV-2 would activate TLR4 directly, probably via its spike protein binding to TLR4 (and/or MD2).

In COVID-19 patients, Sohn et al. recently reported that the expression of TLR4 itself and its downstream signalling mediators were significantly upregulated in peripheral blood mononuclear cells, compared to those in healthy controls [122]. The inflammatory molecules found to be upregulated include CD14, MyD88, Mal (also known as TIRAP), TRAF6, IRAK1, and TICAM (also known as TRIF). These findings strengthen the suggestion of a correlation between increased TLR4 expression and its activation by a component of the SARS-CoV-2 virus, similar to that which occurs in bacterial sepsis. Indeed, bacterial LPS is known to result in significant upregulation of TLR4 expression, in RAW264.7 macrophages for example, along with the DMAP HMGB1 [123]. Furthermore, Kogan et al. report increased TLR4 expression in the myocardium of patients with COVID-19 by immunohistochemistry [124]. Again, this is very similar to that which occurs in bacterial sepsis, as TLR4 expression is known to be significantly elevated in the myocardium upon stimulation with LPS [75, 125].

### 8.3. DAMP-Mediated TLR4 Activation

The other possibility is a secondary mechanism involving damage-associated molecular patterns (DAMPs) released from the lysed or dying cells due to COVID-19, activating TLR4 in the lungs and heart causing inflammation and fibrosis. Host DAMPs were detected in the lungs of patients with SARS-CoV-1 and may play a central role in acute lung injury [71]. A review by Andersson et al. suggests that HMGB1 released as a DAMP or secreted by activated immune cells could activate both TLR4 and the receptor for advanced glycation end-products (RAGE) to generate proinflammatory cytokines [126]. Specifically, the disulphide form of HMGB1 stimulates TLR4, while extracellular HMGB1 can form complexes with DNA, RNA, other DAMP or PAMP molecules; the complexes are endocytosed via RAGE and transported to the endolysosomal system. The endosomes themselves may have TLR4 receptors. HMGB1 at high levels (due to necrosis) would disrupt the endolysosomal system and cause detrimental inflammasome activation, leading to excessive inflammation and lung injury (see [126] and references therein). Interestingly, RAGE is constitutively expressed at high levels in the lungs only, while Andersson et al. referred to a paper by Yang et al. showing that peritoneal macrophages isolated from TLR4 KO mice, but expressing RAGE, produced almost no proinflammatory cytokines in response to HMGB1 stimulation [127]. This shows the importance of the HMGB1/RAGE/TLR4 axis in mediating inflammation [126]. Interestingly, it has also been recently shown that the alarmin S100A9, a DAMP and TLR4 ligand, is a reliable biomarker in severe/critically ill COVID-19 patients [122].

## 9. The Possible Mutual Benefits of Viral Activation of TLR4

Firstly, the question arises as to whether TLR4 activation is beneficial or harmful to the body during an acute viral infection. The answer is probably both. It is necessary for the body to initiate a protective innate immune and inflammatory response against pathogenic viruses/viral components. TLR4 signalling is essential for the secretion of the antiviral cytokine IFN-*β*, which also reduces (cardiac) inflammation and helps control the viral infection [128]. However, if TLR4 activation becomes excessive due to overstimulation whether by viral proteins or by DAMPs released from lysed cells, then it can lead to severe inflammatory consequences and possibly death (e.g., due to the cytokine storm) [110]. If TLR4 is deleted, it can also lead to severe disease and fatal outcomes, due to the inability to mount an immune response. The reader is referred to Figure 1(b) in Olejnik et al. [110], which summarises these links between TLR4 and viral/DAMP activation. Secondly, why does SARS-COV-2 (or other viruses) bind to TLR4 if it initiates interferons against the virus? Fortunately for the human, TLR4 was designed to recognize viral pathogens. For the virus, there could be multiple reasons, as follows:

### 9.1. SARS-CoV-2 Is Likely Using TLR4 to Enter the Cells and/or Increase the Expression of ACE2

Given the remarkable high binding affinity of the spike S glycoprotein of SARS-COV-2 to the TLR4 extracellular domain as determined by biocomputational analyses, the virus could be using TLR4 signalling to increase the expression of ACE2 (or another receptor) through which it would enter the same or neighbouring cells. Cameron et al. found that high interferon *α* and interferon *γ* and strong interferon-stimulated gene (ISG) expression were a novel signature in the early phases of illness from SARS-CoV-1 [129]. Also, IFN-*α*, IFN-*γ*, and TNF-*α* correlated with viral load and were found to be high in patients with severe COVID-19 disease [130]. Zhou et al. found that ISGs are highly expressed in COVID-19 patients, to the point that it exhibits pathogenic potential [31]. Bronchoalveolar lavage (BAL) fluid taken from SARS-CoV-2-positive patients was found to have a robust immunopathological ISG expression, compared to that from community-acquired pneumonia patients (of other viral and bacterial causes) and healthy controls. However, it appears sampling timepoints and admission to the intensive care unit were variable among the COVID-19 patients—diagnosed from around 4 to 15 days after symptom onset. Therefore, it may be difficult to ascertain a correlation with disease severity for that particular study, although Broggi et al. also reported that BAL of patients with severe COVID-19 disease presented with high expression of type I and type III interferons [131]. Ziegler et al. have shown that ACE2 is a human ISG *in vitro* using human airway epithelial cells and extended it to *in vivo* viral infections of influenza A and B [32]. This is reasonable because ACE2 is anti-inflammatory, and the TLR4 alternative interferon-ISG pathway expresses anti-inflammatory cytokines. Therefore, SARS-CoV-2 may benefit from TLR4 activation to increase ACE2 expression in order to enter into the cells. Indeed, a similar mechanism is employed by the Coxsackievirus B3 where TLR4 signalling via the MyD88 pathway leads to increased Coxsackie-adenovirus receptor (CAR) expression which is responsible for virus internalisation, leading to increased viral entry and proliferation in the cardiomyocytes [132, 133]. In fact, viral concentrations of Coxsackievirus group B serotype 3 (CVB3) in the hearts were significantly decreased in MyD88-negative mice [134], which means that there was less entry of CVB into the myocardial cells. It is also employed by the MMTV whereby the virus directly binds TLR4 on dendritic cells to upregulate its entry receptor CD71 on these cells *in vitro* and *in vivo* [135]. Furthermore, by binding to TLR4 and activating the alternative TRIF/TRAM pathway, the virus may even be exploiting the movement of the receptor itself to gain entry into the cell, since TLR4 becomes internalised into endosomes in the alternative pathway (see [22]); i.e., TLR4 may be involved in receptor-mediated endocytosis of SARS-COV-2. Finally, another possibility could be the structural membrane M protein binding to TLR4.

### 9.2. SARS-CoV-2 Virus May Alter the Balance towards MyD88-Dependent Signalling Rather than the TRIF/TRAM-Dependent Antiviral and Anti-Inflammatory Interferons, Thereby Causing Myocarditis and Lung Injury

The SARS-CoV-2 virus may lead to aberrant TLR4 downstream cellular signalling to cause cardiac injury (viral myocarditis) and lung injury, by altering the balance between MyD88-dependent and independent signalling, given the role of TLR4 in mediating abnormal inflammation and pathological fibrosis. Such abnormal or unregulated signalling relating to interferons and IFN-stimulated gene (ISG) expression was observed in patients who succumbed to SARS-CoV-1. Cameron et al. found that SARS-CoV-1 patients with poor outcomes had deviated ISG expression, persistent chemokine levels, and deficient antibody production in in the later phases, suggesting a malfunction of the switch from innate to adaptive immunity [129]. Recent studies have found that the production of IL-1*β*, IL-18, TNF-*α*, and IL-6 was high in severe COVID-19 patients especially those with severe respiratory failure and are linked to the cytokine storm [130, 136]; these cytokines are downstream of the TLR4 canonical MyD88-dependent pathway. Ruan et al. indicated that “fulminant myocarditis” or sudden myocarditis (characterised by a severe disease and quick progress) and a virus-activated “cytokine storm” syndrome are causes of death in COVID-19 [40].

Many viruses can cause viral myocarditis including the influenza virus [137], and viral infection is thought to be the leading cause of myocarditis, a condition which leads to dilated cardiomyopathy [138]. An important example showing that viruses cause viral myocarditis through TLR4 signalling is that of the Coxsackievirus B3 (CVB3), reviewed by Yang et al. [89]. CVB3 infection increases the levels of the proinflammatory cytokines IL-1*β* and IL-18 in wild-type mouse hearts, but not in TLR4-deficient mice [89, 139]. Furthermore, TLR4-deficient mice had significantly less myocarditis and more resistance to CVB3 infection with less viral replication than wild-type mice [139]. Viral myocarditis appears to be driven more by aberrant MyD88-dependent signalling rather than the alternative TRIF/TRAM pathway [89]. Cardiac MyD88 protein levels were significantly increased in the hearts of wild-type mice exposed to CVB3 [134]. MyD88-negative mice had decreased production of the proinflammatory cytokines IL-1*β* and IL-18, but the balance was shifted towards more direct expression of IRF3 and IFN-*β*, and thus, these mice had a dramatically higher survival rate than wild-type mice [134]. Supporting this is the study by Riad et al. which showed that TRIF-negative mice that were infected with CVB3 had suppression of IFN-*β* (TLR4 dependent), worse cardiac remodelling, severe heart failure, and 100% mortality [128]. In fact, treatment of the TRIF-negative CVB3-infected mice with murine IFN-*β* resulted in reduced cardiac inflammation and improved virus control [128]. Thus, TLR4 appears to have 2 pathway arms whose balance is dysregulated in viral infections: one predominantly proinflammatory that exacerbates myocarditis (MyD88 dependent) and the other anti-inflammatory and anti-viral (MyD88 independent).

Furthermore, the idea of spatial (tissue specific) and temporal (time specific) variations in TLR4 signalling pathways could be occurring, whereby the SARS-CoV-2 would cause the activation of one pathway rather than the other depending on the cell type where TLR4 resides and/or the stage of infection. In contrast to high interferon I levels in the lungs of COVID-19 patients with severe disease [131], Hadjadj et al. found that peripheral blood immune cells in patients with severe or critical COVID-19 disease had diminished type I interferon response (absent IFN-*β* and low IFN-*α*), but enhanced proinflammatory cytokines (IL-6 and TNF-*α* production in the blood) [140].

### 9.3. TLR4 Signalling Also Activates PI3k Phosphatidylinositol 3-Kinase (PI3K), a Cell Survival Factor

PI3K is a cell survival factor that prevents premature apoptotic cell death during viral infections, including SARS-CoV-1 (see [110, 141] and references therein). Therefore, the virus is essentially “buying time” to replicate inside the cells. In fact, Li et al. showed that glucan phosphate, a TLR4 ligand, causes a shift of TLR4 signalling from the predominant NF-*κ*B pathway to the PI3K-Akt pathway which promotes cell survival, inhibits apoptosis, and serves a protective role in myocardial I/R injury [109]. Note that the spike protein of SARS-CoV-2 is a glycoprotein having sugar moieties, which therefore may have a similar effect to that of glucan phosphate. This also serves a simultaneous beneficial effect to the cell, preventing its premature death. Hence, this pathway may confer a protective role in myocardial inflammation [89], although it is advantageous for the virus.

## 10. The Role of Pulmonary Surfactants and TLR4 in the Lung

Studies have shown that pulmonary surfactant phospholipids which line the alveolar compartment at the air/liquid interface of the tissue are antagonists of TLR4 (and TLR2) in the lung [33, 37]. Kuronuma et al. had demonstrated that palmitoyl-oleoyl-phosphatidylglycerol (POPG) inhibits the phosphorylation of several downstream proteins in the TLR4 signalling cascade [37], while Voelker and Numata showed that phosphatidylinositol (PI) is nearly as potent as POPG, and that POPG and phosphatidylinositol (PI) inhibit the secretion of TNF-*α* in the presence of LPS [33]. This has the physiological advantages of preventing unwanted inflammation when exposed routinely to environmental stimuli such as microbial particles and “ambient levels of airborne LPS” [33, 142]. This prevents unnecessary interference with gaseous exchange, making a high threshold for initiating proinflammatory pathways until TLR4 activation is warranted [33]. Surprisingly, POPG and PI dramatically prevent and curtail RSV and IAV viral infection in mice. The lung histology images in Voelker and Numata are remarkable; sections from mice infected with the virus and treated with POPG or phosphatidylglycerol (PG) were comparable to the sections from control, uninfected mice. Histopathology scores of the lungs infected with the virus and receiving POPG were almost equivalent to sham-infected mice [33].

The studies showed that POPG acts as a competitive ligand for the LPS-binding sites on CD14 and MD2 (the coreceptors for TLR4) and hence inhibit the extensive inflammatory processes associated with those viral infections [33, 37]. The authors also state that the mechanism of action of the lipids is “disruption of virus particle binding to the host cell plasma membrane receptors, required for viral uptake” [33], without mentioning which receptors. However, in the case of SARS-CoV-2, it is likely to be TLR4 in addition to ACE2.

This has raised the possibility of POPG and PI being given as a treatment to subjects with active viral infection to prevent infections of naïve cells [33]. Furthermore, this confirms that these pulmonary surfactant phospholipids are shielding the alveolar cells from TLR4, the possible receptor involved in entry for viruses or the powerful inflammatory receptor for the cells.

## 11. A Model for COVID-19

We propose that SARS-CoV-2 first infects ACE2-positive type II alveolar cells in the lungs that are responsible for producing pulmonary surfactants, entering the cells through its spike protein binding with the ACE2 receptor. The infected type II alveolar cells are destroyed and lysed. This leads to the profound decrease in the production of pulmonary surfactants in the alveoli, affecting the air/tissue surface tension, as well as exposing the TLR4 extracellular binding sites on the alveolar and bronchial epithelial cells. The high surface tension affects lung compliance (alveolar expansion) and compromises breathing. The virus then binds TLR4 on the other alveolar and bronchial epithelial cells via its spike (S) protein or other capsid protein, using it to either (a) increase the expression of ACE2 via interferons and ISGs, (b) directly enter the cell using TLR4, (c) cause aberrant TLR4 signalling towards the MyD88-dependent proinflammatory pathway rather than the TRIF/TRAM-dependent interferon pathway, and/or (d) activate PI3K signalling in infected cells to prevent apoptosis. Infiltration of inflammatory cells leads to the upregulation of TLR4 on those lung cells, and the activation of TLR4 on inflammatory cells occurs, which is biased towards MyD88-dependent acute inflammatory signalling, further exacerbating the situation. By binding TLR4, SARS-CoV-2 triggers rampant inflammation and ALI, and as it enters the blood circulation, it infects the heart via ACE2 and/or TLR4 (causing myocarditis), as well other organs such as the skin, kidneys, and GI tract where TLR4 is expressed. Moreover, HMGB1 and other DAMPS released from necrotic/lytic cells, as well as ambient LPS levels entering the lungs or released from opportunistic gram-negative bacteria, can also activate TLR4, amplifying the already severe inflammation. LPS-TLR4 signalling also activates the NLRP3 inflammasome, leading to further IL-1*β* release [106].

As more alveolar cells are infected and destroyed, the sharp drop in pulmonary surfactants continues, and acute respiratory distress syndrome (ARDS) follows. All the above lead to excessive inflammation and severe disease, including sepsis and a possible cytokine storm. TLR4 activation on platelets by the virus (viraemia) or DAMPs increases the risk of thrombosis, manifesting in the form of myocardial infarctions and embolisms. Additional evidence supporting this model is that a corticosteroid, dexamethasone, is effective at reducing 28-day mortality from COVID-19 among those receiving invasive mechanical ventilation or oxygen at randomization [143]. Corticosteroids suppress NF-*κ*B and AP-1 proinflammatory transcription factors [144], which are downstream targets of signalling from TLR4 and other TLRs as well. Furthermore, with time, overstimulation of TLR4 on structural fibroblasts in the heart and lungs can lead to fibrosis [18, 78]. These events are summarised in Figure 1 (graphical abstract).

## 12. Possible Therapeutic Options for Treating COVID-19 which Target TLR4

### 12.1. Pulmonary Surfactants by Intratracheal Administration, for the Lungs

Based on the studies reviewed in here, the testing of pulmonary surfactants in clinical trials as a potential treatment for the lungs of infected individuals with SARS-CoV-2 is plausible and several recent trials have been initiated. This would potentially serve 3 simultaneous benefits: (a) it would increase the compliance of the lung alveoli and prevent their collapse; (b) confer antiviral actions by shielding and preventing infection of naïve cells, especially if TLR4 is proven to be an entry receptor or contributes to ACE2 upregulation; and (c) block TLR4 to reduce inflammation and excessive cytokine production.

Lung surfactants are normally given to preterm new-born babies who are born prematurely, to treat neonatal respiratory distress syndrome (NRDS), also known as hyaline membrane disease. The ARDS seen in COVID-19 patients shares many features with NRDS especially the formation of hyaline membranes [41]. Some of the pulmonary surfactants available include [145–147] bovine or calf pulmonary surfactants, containing mainly Beractant (brands: Beractant®, Survanta®, and Beraksurf®) or Calfactant (brands: Calfactant® and Infasurf®), respectively, and porcine pulmonary surfactants, containing mainly Poractant Alfa (Curosurf®). There are also synthetic lung surfactants containing mainly Colfosceril palmitate, also known as dipalmitoyl phosphatidylcholine or “DPPC,” which is the major natural surfactant (brands: Exosurf Neonatal®, Lucinactant®, and Surfaxin®); but it appears these are discontinued or no longer available to treat RDS [147]. To our knowledge, there are currently five recently registered clinical trials, which are investigating pulmonary surfactant therapy in ARDS of COVID-19 that have been added to the Clinical Trialshttp://Trials.gov database [148]. Of these, only one is currently recruiting (NCT04384731). Four trials were not yet recruiting as on 28/08/2020 (NCT04375735, NCT04389671, NCT04362059, and NCT04502433). These are based on the known effect of pulmonary surfactants in improving lung function and survival in NRDS.

### 12.2. Targeting TLR4 through TLR4 Antagonists and Signalling Inhibitors

Agonists and antagonists of TLRs have previously been proposed to have utility as vaccine adjuvants or antiviral compounds ([113] and references therein). The body needs a certain amount of TLR4 stimulation to fight the virus, but not an overstimulation, especially in the later stages. However, other TLRs will be able to produce some interferons if TLR4 is antagonised. For example, the endosomal TLR7 that recognises single-stranded RNA from viruses also induces type I interferon production but via MyD88-dependent pathways [149]. Furthermore, the endosomal TLR9 recognises unmethylated cytosine-phosphate-guanine (CpG) dinucleotides, which are present in many bacteria, DNA viruses, and mitochondrial DNA (DAMP) released from dying cells [85, 90]. Activation of TLR9 results in MyD88-dependent inflammatory signalling [90], which may be of some relevance in COVID-19 when host-derived DNA is released due to tissue damage or if there are opportunistic bacterial infections. Kitazume-Taneike et al. showed that ablation of TLR9 attenuates myocardial ischaemia/reperfusion injury and inflammatory responses in mice, which is relevant since COVID-19 can lead to myocardial ischaemia and therefore tissue damage [150]. This also supports the case for TLR4 antagonism from a tissue damage and DAMP viewpoint, aside from its proposed involvement in SARS-CoV-2 cell entry. As mentioned previously, TLR4 is present on both the cell surface and in endosomes. Based on the evidence presented in this review, targeting TLR4 could be effective in treating COVID-19.

Recently, based on Choudhury and Mukherjee [30], Patra et al. also suggested targeting TLRs in general to combat COVID-19 [151]. However, they did not explicitly propose the use of TLR4 antagonists *per se* against SARS-CoV-2. However, they do mention that some TLR4 antagonists including Eritoran and TAK242 (both proposed here) were used successfully in the treatment of inflammation associated with cancer, rheumatoid arthritis, and other inflammatory diseases ([151] and references therein). Based on our proposal (see Disclosure: DOI) and Choudhury and Mukherjee [30], another recent report speculates on the hypothesis that the TLR4 signalling pathway provides opportunities for the targeting of TLR4 by antagonists in severe COVID-19 patients with cardiometabolic complications provided that the involvement of TLR4 is proven [152]. Based on the comprehensive and detailed evidence reviewed here, we support in principle the trial of TLR4 antagonists in the treatment of severe COVID-19 infections to (1) reduce the severe inflammation, particularly in the lungs and heart, but also systemically (2) block the entry mechanism involving ACE2 upregulation that we have proposed. Some further studies relevant to the use of TLR4 antagonists are highlighted below:

#### 12.2.1. The TLR4 Antagonists: Eritoran and Resatorvid

TLR4 antagonist given in the later severe stages of COVID-19 could mitigate against the aberrant excessive TLR4 stimulation that may lead to a cytokine storm. Furthermore, it may block entry of the virus earlier on. Eritoran has been studied in an antiviral context, and it protected mice from lethal influenza infection and ameliorated influenza-induced lung injury in mice by inhibiting the cytokine storm [153]. Additionally, it inhibited RSVF-, DENV-NS1-, and EBOV glycoprotein-mediated TLR4 activation ([110] and references therein). Furthermore, it blocked HMGB1-mediated TLR4-dependent signalling *in vitro* and circulating HMGB1 *in vivo* [112]. Moreover, it has been reported *in vivo* to reduce both murine cardiac hypertrophy and myocardial ischaemia/reperfusion injury, by favouring the alternative anti-inflammatory pathway (IL-10) over the MyD88-dependent pathways, thereby suppressing the production of proinflammatory cytokines (IL-1*β*, IL-6, and TNF-*α*) [154, 155]. Therefore, it will be useful to reduce myocardial injury associated with COVID-19 and serve multiple roles systemically. However, although Eritoran as a treatment for sepsis was well-tolerated, it did not reduce systemic inflammation or 28-day mortality in phase II and III clinical trials, respectively (see [22, 89] and references therein). However, this was against LPS-induced sepsis, not viral sepsis. For clinical trials pertaining to the use of Eritoran in LPS-induced sepsis and possible reasons for the negative outcomes, the reader is referred to other reviews [22, 89].

Resatorvid (also known as CLI-095 or TAK-242) is a small-molecule-specific inhibitor of TLR4 (signalling), by selectively binding to the TLR4 intracellular domain, effectively disrupting the protein-protein interactions between TLR4 and its adaptors, whether MAL (also known as TIRAP) or TRAM [156]. Therefore, this inhibits both NF-*κ*B and interferon-sensitive response elements [156]. Resatorvid significantly reduced CD4+ T lymphocyte cell death induced by the Ebola virus glycoprotein, which binds TLR4 [157]. In mice who were administered intratracheal LPS, intravenous Resatorvid effectively reduced the production of inflammatory mediators and the increase in lung permeability as well as neutrophil accumulation, thus providing promise in treating ALI and pneumonia caused by gram-negative bacterial infections [158]. It also inhibits TLR4/MyD88/NF-*κ*B signalling and reduces NLRP3 inflammasome activation in rat cardiomyocytes, while reducing myocardial injury and improving cardiac function after coronary microembolisation *in vivo* [159]. However, in a phase II clinical trial, it failed to suppress cytokine levels in patients with sepsis and shock or respiratory failure, although mortality in the treated group was lower but not significant [22, 160]. Again, this may not be entirely relevant as it was primarily LPS-induced sepsis, not viral sepsis. For further information on Resatorvid, the reader is referred to a review by Kuzmich et al. [22].

#### 12.2.2. Glycyrrhizin

The potential use of glycyrrhizin for COVID-19 has been reviewed in detail by Murck et al. [161] and Andersson et al. [126]. Glycyrrhizin is an active ingredient extracted from liquorice plant and has been used in traditional Chinese medicines to control COVID-19. It gets metabolized in the human gut to the systemically active metabolite glycyrrhetinic acid [161]. It appears to possess direct antiviral properties and was reported to inhibit *in vitro* replication and penetration of SARS-CoV-1 [126, 161, 162]. More importantly, it has an anti-inflammatory effect through downregulation of HMGB1-mediated inflammation and TLR4 antagonism (see [126, 161] and references therein). Given its dual antiviral effects and TLR4 antagonism, as well as being a natural compound, it may be a promising candidate in the treatment of COVID-19.

#### 12.2.3. Nifuroxazide

Nifuroxazide is an oral nitrofuran antibiotic that is already licensed (FDA-approved) and used to treat diarrhoea. Khodir et al. did a preclinical study using nifuroxazide in sepsis (LPS)-associated multiorgan injury, which is relevant since sepsis accounts for a high percentage of death in COVID-19 [75]. The study showed that nifuroxazide attenuates LPS-associated ALI and myocarditis when administered to mice either in a curative regimen (LPS injection then after 24 hours nifuroxazide for 7 days) or a prophylactic regimen (nifuroxazide for 10 days, then LPS injection). Note that mice were injected with LPS, not bacteria. The mechanism of action of nifuroxazide was shown to be via interruption of TLR4-NLRP3/IL-1*β* signalling [75]. Furthermore, while LPS increased TLR4 content in the lungs and heart by at least twofold, nifuroxazide was shown to significantly reduce TLR4 content by 45.7% in the lungs and 31.2% in the heart in the curative regimen and more so in the prophylactic regimen, compared to the LPS control [75]. Nifuroxazide has also shown anti-inflammatory potential by inhibiting STAT3, for example in diabetes-induced nephropathy in rats [163], and in attenuating acute graft vs. host disease in mice [164]. In particular, expression levels of TNF-*α* and IL-18 in diabetic renal tissue were reduced [163]. IL-18 is also induced by the NLRP3 inflammasome in the lungs and heart. The above evidence shows that nifuroxazide may be of benefit in critically ill patients with sepsis in COVID-19, but clinical trials are currently lacking.

## Abbreviations

ACE2: Angiotensin converting enzyme 2
ALI: Acute lung injury
Akt: Protein kinase B
AP-1: Activator protein-1
ARDS: Acute respiratory distress syndrome
CD14: Cluster of differentiation 14
COVID-19: Coronavirus disease-2019
DAMP: Damage-associated molecular pattern
HMGB1: High mobility group box 1
IFN: Interferon
I*κ*B: Inhibitory *κ*B
IL: Interleukin
IRAK: Interleukin-1 receptor-associated kinase
IRF: Interferon regulatory factor
ISGs: Interferon-stimulated genes
LPS: Lipopolysaccharide
MAL: MyD88 adaptor-like protein
MD2: Myeloid differentiation factor 2
MyD88: Myeloid differentiation factor 88
NF-*κ*B: Nuclear factor-*κ*B
NRDS: Neonatal respiratory distress syndrome
SARS-CoV-1 (or SARS-CoV): Severe acute respiratory syndrome-associated coronavirus-1
SARS-CoV-2: Severe acute respiratory syndrome-associated coronavirus-2
TAB2: TAK1-binding protein 2
TAK1: Transforming growth factor-*β*-activated kinase 1
TIR domain: Toll/interleukin-1 receptor domain
TIRAP: TIR domain-containing adaptor protein
TLR: Toll-like receptor
TLR4: Toll-like receptor 4
TNF-*α*: Tumour necrosis factor-*α*
TRIF: TIR-domain containing adapter-inducing IFN-*β*
TRAF: Tumour necrosis factor receptor-associated factor
TRAM: TRIF-related adaptor molecule
PAMP: Pathogen-associated molecular pattern
PI3K: Phosphatidylinositol 3-kinase.
